# Study on Molecularly Imprinted Polymers Obtained Sonochemically for the Determination of Aflatoxins in Food

**DOI:** 10.3390/molecules28020703

**Published:** 2023-01-10

**Authors:** Sara Palmieri, Dounia Elfadil, Federico Fanti, Flavio Della Pelle, Manuel Sergi, Aziz Amine, Dario Compagnone

**Affiliations:** 1 Department of Bioscience and Technology for Food, Agriculture and Environment, University of Teramo, via Renato Balzarini 1, 64100 Teramo, Italy; 2 Laboratory of Process Engineering and Environment, Faculty of Sciences and Techniques, Hassan II University of Casablanca, Mohammedia 20650, Morocco

**Keywords:** aflatoxins, food samples, HPLC–MS/MS analysis, molecularly imprinted polymers, mycotoxins extraction

## Abstract

Aflatoxins (AFs) are fungi secondary metabolites produced by the Aspergillus family. These compounds can enter the food chain through food contamination, representing a risk to human health. Commercial immunoaffinity columns are widely used for the extraction and cleanup of AFs from food samples; however, their high cost and large solvent consumption create a need for alternative strategies. In this work, an alternative strategy for producing molecularly imprinted polymers (MIPs) was proposed to extract aflatoxins AFB1, AFB2, AFG1, and AFG2 from complex food samples, using liquid chromatography coupled with tandem mass spectrometry (LC–MS/MS). The MIPs were synthesized via a low-cost and rapid (5 min) sonochemical free-radical polymerization, using 1-hydroxy-2-naphthoic acid as a dummy template. MIPs-based solid phase extraction performance was tested on 17 dietary supplements (vegetables, fruits, and cereals), obtaining appreciable recovery rates (65–90%) and good reproducibility (RSD ≤ 6%, n = 3); the selectivity towards other mycotoxins was proved and the data obtained compared with commercial immunoaffinity columns. The proposed strategy can be considered an alternative affordable approach to the classical immunoaffinity columns, since it is more selective and better performing.

## 1. Introduction

Aflatoxins (AFs) are a group of compounds having as basic structure a coumarin and a double-furan ring and are produced as secondary metabolites by fungi of Aspergillus family as *A. flavus, A. parasiticus,* and *A. nomius* [1,2,3]. AFs can contaminate foodstuffs such as cereals, nuts, fruits, dried fruits, and oilseeds in field (harvest period) or during drying/storage [4,5,6,7]. The ingestion of large amounts of AFs over a short period can cause vomiting, abdominal pain, swelling, jaundice, acute liver damage, and even death; on the other hand, long-period ingestion of low amounts of AFs is also considered a significant risk for health [8,9]. Despite more than twenty AFs having been identified, only aflatoxin G1 (AFG1), aflatoxin G2 (AFG2), aflatoxin B1 (AFB1), and aflatoxin B2 (AFB2) are classified as toxic to human health [10]. To reduce health risks, European (EU) legislation has set the maximum levels of AFG1, AFG2, AFB1, and AFB2 for processed food at 0.2 µg/kg [11].

Different analytical techniques can be employed for the determination of AFs in various samples [12,13]. These techniques include thin layer chromatography, capillary electrophoresis, gas chromatography (GC) coupled with mass spectrometry (MS) [2], and high-performance liquid chromatography (HPLC) coupled with fluorescence or [14] MS detection [15], all of which have been widely used. Regardless of the detection technique employed, the real analytical issue relies on the selective extraction of AFs from complex matrices; for this reason, the search for extraction strategies for AFs is still a significant research topic. Currently, solid phase extraction (SPE) cartridges for the isolation of mycotoxins including AFs are based mainly on immunoaffinity columns (IAC), which allows effective sample cleanup [16,17]. This approach has been adopted as an official method by AOAC International [18], but still has different drawbacks, such as high cartridge cost (EUR 10–13 per immunoaffinity column), time-spending procedures, and large consumption of solvents.

In the last few years, there has been an exponential growth in research works regarding the development of molecularly imprinted polymers (MIPs) for mycotoxins analysis [19,20]. The effectiveness of MIPs relies on the formation of nanostructured binding sites in organic polymers, which are obtained using different strategies. MIPs are robust alternatives to antibodies which, although they often have a higher affinity for target molecules, may have different drawbacks, including short shelf life (6–12 months), a narrow working pH range, limited reuse, high cost, ethical issues linked to their production, and specific storage requirements [21]. On the other hand, MIPs are fully synthetic and animal-free alternatives. MIPs are achieved by synthesizing a polymer in the presence of a given template molecule, followed by a template removal step; this allows the structural characteristics of the target molecule in terms of shape, size, and functionality to be imprinted onto the resulting polymer [19]. Properly designed and synthesized MIPs can selectively capture molecules with different structures, from small molecules to large organic molecules. Given their peculiar features, MIPs are considered smart and affordable materials for separation, owing to their thermochemical stability, low cost, reusability, and effectiveness even in complex matrices. For these reasons, MIPs have largely been used as adsorbent phases for SPE cartridges as well as being successfully used for different analytical applications [19,22].

Different routes have been employed to produce MIPs, exploiting different approaches to promote polymerization, such as thermal heating [23,24], photoinduction, microwave [25], and sonication in ultrasonic baths [26,27]. In the last few years, researchers have explored the rapid synthesis of MIPs by high-power ultrasound probes, which enables the speeding up of the polymerization process [28,29,30]; this strategy has also allowed the production of effective MIPs for analytes in those cases where conventional techniques were not successful. To the best of our knowledge, there are only a few works regarding MIPs for AFs and the proposed strategies are based on long procedures (Table S1). On the other hand, nothing is reported regarding fast sonochemical approaches.

This work proposes a rapid (5 min) sonochemical free-radical synthesis of MIPs for AFs (AFB1, AFB2, AFG1, and AFG2), using 1-hydroxy-2-naphthoic acid as a cheap and non- toxic dummy template. The MIPs was used as the sorbent phase in SPE cartridges, and the performance was tested analyzing AFs in seventeen dietary supplements (vegetables, fruits, and cereals) in conjunction with LC–MS/MS. The selectivity towards other mycotoxins was evaluated and the performance of MIPs was compared to immunoaffinity columns.

## 2. Results and Discussion

### 2.1. Monomer Selection

The rapid synthesis of MIPs for AFs (in 5 min) relies on the employment of a high-power ultrasound probe (see Section 3.3); this strategy significantly speeds up the polymerization process and simultaneously returns adequate MIP structures. Compared with other methods, this approach allows for accelerating chemical reactions, lowering the requirement for reagents, and initiating polymerization. To the best of our knowledge, the synthesis of MIPs for AFs has so far been carried out using long-time procedures (see Supplementary Materials Table S1).

**Figure 1:**
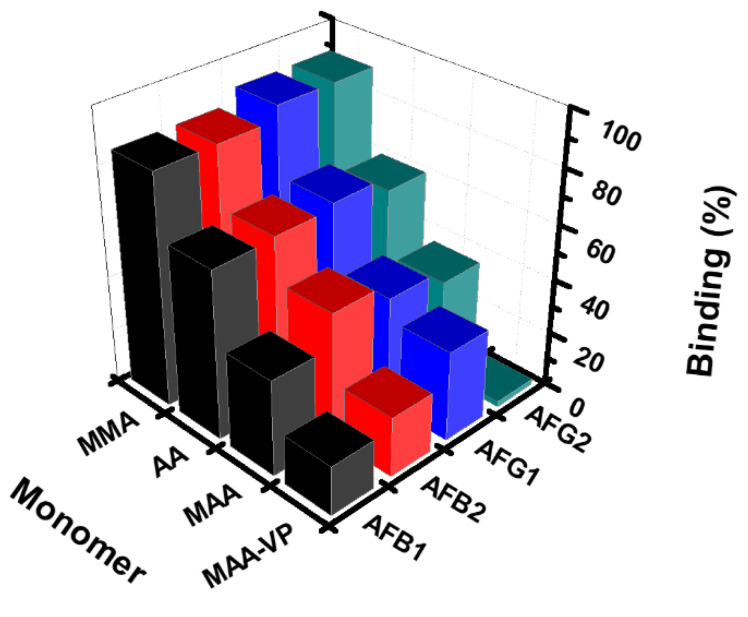
3D histogram of % binding capacities of MIPs towards aflatoxin B1 (black), aflatoxin B2 (red), aflatoxin G1 (blue), aflatoxin G2 (green) at a concentration of 5 µg/mL. MIPs were synthetized with different monomers: methacrylamide (MMA), acrylamide (AA), methacrylic acid (MAA), and methacrylic acid +2-vinylpyridine (MAA-VP).

Thus, to obtain an effective MIP within a fast timeframe, a study of the functional monomer was performed. To this aim, we tested acrylamide (AA), methacrylic acid (MAA), methacrylamide (MMA), and methacrylic acid +2-vinylpyridine (MAA-VP) in DMSO, using ethylene glycol dimethacrylate (EGDMA) as the cross-linker and 2,2 azobis-isobutyronitrile (AIBN) as the initiator. The AFs structures that were taken into consideration, along with the monomers and the dummy template, are depicted in Figure S1. As reported in Figure 1, MMA-based MIP yielded the best result, considering the adsorption capacity of AFs (80–95%); the selection was also confirmed by the higher imprinting factor obtained (see Section 2.3.).

Among the few works present in the literature, the monomers MAA and AA are usually employed for the synthesis of MIPs for AFs; in these works, the dummy template used is 3-coumarincarboxylate [4,5,6]. In our case, naphthoic acid was employed as the dummy template because of the low cost, solubility in DMSO, and its previously reported ability to interact with MMA [31,32]. Naphthoic acid, thanks to the conjugated aromatic ring, yields rigid results and mimics the core structure of the AFs; at the same time, the quantitative removal from the formed polymer was easily attained. Naphthoic acid, unlike 3-coumarincarboxylate, does not possess oxygen intercalated in the aromatic rings, which allows it to mimic approximately all the 4 targeted AFs; the latter hypothesis was confirmed by the similar binding capacity obtained for the 4 studied AFs (Figure 1).

### 2.2. Optimization of MIPs–SPE

In order to develop an effective and selective AFs extraction procedure, the parameters of the MIPs-based SPE extraction were carefully optimized (Table S2); the tests were carried out in parallel for both MIPs and NIPs. The first step was selecting the best conditions for MIP adsorption; to this end, the solvents used in the different steps and the amount of polymer were both optimized. The ‘solvent’ used in the loading is crucial for the adsorption of the analyte by MIPs; in accordance with the literature, water resulted in the best solvent for AFs adsorption [1,6]. The maximum adsorption was reached using 5 mg of polymer in the 1 mL SPE cartridges. Lower amounts gave lower retention, while a high MIP amount did not provide any further enhancement of AFs adsorption. To reduce possible interferences a washing step was introduced; water containing 0.5% ACN resulted in the best washing phase among those that were tested. The 2% acetic acid in methanol allowed for the quantitative release of the retained AFs. The reusability of the MIPs was also evaluated as being a relevant factor in practical applications in terms of cost-saving; the MIPs may be used up to 3 times without a significant decrease in binding capacity (RSD 5%, n = 3).

### 2.3. MIP Performance

**Figure 2:**
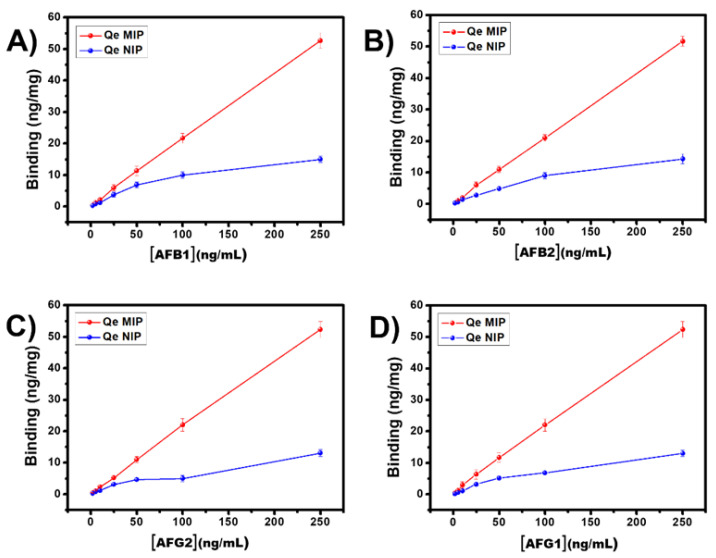
Adsorption capacity (%) of the MIPs (red) and NIPs (blue) obtained analyzing different aflatoxin amounts (from 2 to 250 ng/mL). Graphs (**A**–**D**) correspond to aflatoxins B1, B2, G1, and G2, respectively.

The binding adsorption capacities of MIPs and NIPs for the 4 AFs studied (i.e., AFB1, AFB2, AFG1, and AFG2) were systematically investigated as a function of the initial AFs amount (Figure 2).

Under increasing concentrations of AFs, the AFs uptake of the MIP was significantly higher as compared with NIP; this indicates that the MIP possesses a higher retention capacity than NIP, conferred by its imprinted cavities. The outstanding reproducibility obtained for all concentrations of AFs studied (RSD ≤ 2%, n = 3) endorses the MIPs–SPE procedure feasibility. The imprinting factors (IF) obtained were 3.5 ± 0.02, 3.6 ± 0.04, 4 ± 0.05, 4.2 ± 0.03, for AFB1, AFB2, AFG2, and AFG1, respectively, demonstrating the superior performance of the MIPs compare to NIPs. This occurs because the MIPs possess multi-electrostatic interaction sites and spatial structures that recognize the AFs molecules, while the NIPs give rise only to non-specific adsorption. The equilibrium data were fitted using the Langmuir and Freundlich isotherms [25], and the obtained parameters and curves are reported in Table 1 and Figure S2, respectively.

Comparing the regression coefficients (R^2^) obtained, Langmuir is the most adequate model for interpreting the adsorption of AFs onto MIPs and NIPs; this demonstrates how the adsorption process of the MIPs for the AFs (AFB1, AFB2, AFG1, AFG2) occurs in a monolayer homogeneous surface. According to the Langmuir isotherm model, the maximum adsorption capacities (Qmax) of the MIPs for the AFs are 50.46 (AFB1), 49.94 (AFB2), 49.47 (AFG1), and 48.21 (AFG2) ng/mg; meanwhile for the NIPs, they are 16.72 (AFB1), 16.78 (AFB2), 27.03 (AFG1), and 17.35 (AFG2) ng/mg.

### 2.4. MIP Selectivity

**Figure 3:**
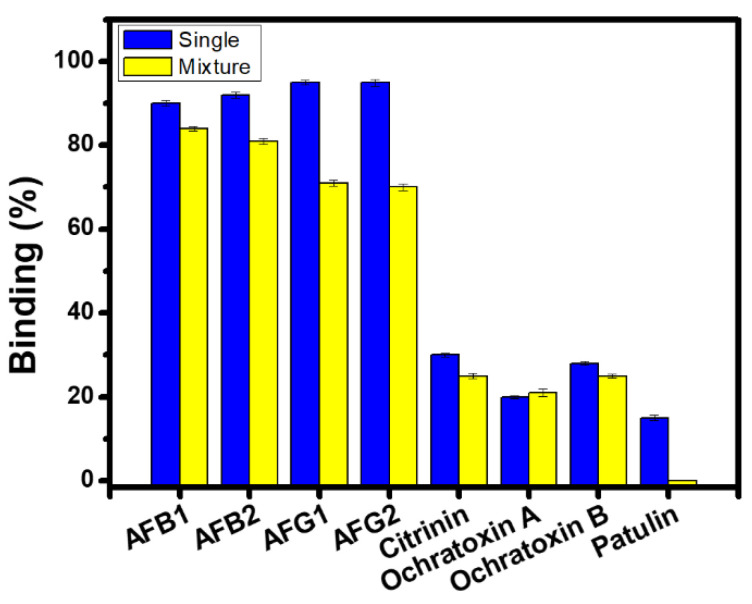
Selective adsorption of MIPs for single compounds in blue (AFB1, AFB2, AFG1, AFG2, Citrinin, Ochratoxin A, Ochratoxin B, and Patulin) and in a mixture, with the compounds in yellow at a concentration of 5 ng/mL.

The MIP selectivity and the potential competition for binding sites have been studied by analyzing potential interference molecules belonging to the mycotoxin class i.e., Citrinin, Ochratoxin A, Ochratoxin B, and Patulin; the study was conducted both in single compounds and in a mixture test with the four aflatoxins and other mycotoxins (AFB1, AFB2, AFG1, AFG2, Citrinin, Ochratoxin A, Ochratoxin B, and Patulin) at a concentration of 5 ng/mL. The test was conducted following the procedure reported in Section 3.5 and the eluates were analyzed by LC–MS/MS. In Figure 3, the data obtained is expressed as a binding percentage (%), which pointed out a significant selectivity towards AFs. The retention values of the non-AFs mycotoxins fell below 30% in the presence of one or a mixture of toxins.

In the presence of a mixture of toxins (Figure 3), the retention of AFs decreases slightly probably due to non-selective interactions of other mycotoxins. However, this reduced retention can be treated as negligible considering the very low probability of the occurrence of the mycotoxins at this high amount in real samples.

### 2.5. Method Validation

The developed analytical procedure based on MIPs–SPE and LC–MS/MS was then validated according to FDA guidelines [26], in terms of linearity, detection and quantification limits and precision. Linear calibrations were constructed for all AFs in the range LLOQs–50 ng/mL; determination coefficients ≥ 0.99 were obtained for all AFs as shown in Table S3. The lower limit of detection (LLOD) and lower limit of quantification (LLOQ) were calculated based on S/N = 3 and S/N = 10, respectively, from the chromatograms of the calibration curves; the calculated LLODs and LLOQs are shown in Table S3.

The accuracy of the method, expressed as BIAS %, was estimated by spiking blank samples (n = 3) at three levels (0.2, 5, and 10 µg/kg); the obtained values, within +5%, demonstrate good performance of the method over the entire calibration range (see Table S4).

To estimate the precision, 3 replicates of the extraction experiment were carried out at 0.2, 5, and 10 µg/kg for AFB1, AFB2, AFG1, and AFG2 on the same day (intra-day), and on different days (inter-day); RSD ≤ 6% were obtained (see Table S5).

Good recoveries (65–90%; RSD ≤ 6%, n = 3) were obtained for all targeted analytes at the three studied levels (0.2, 5, and 10 µg/kg). Moreover, the matrix effect was evaluated for all tested food supplements; ionization enhancement and suppression were limited for all the tested concentration levels (RSDs ≤ 7%).

### 2.6. Sample Analysis and Comparison with Immunoaffinity Columns

Sample analysis was carried out on 17 heterogeneous food supplements i.e., Ginger, Echinacea purpurea, ginseng, hypericum, red elm, saffron, mango, red rice, parsley, red fruits, grapefruit, magnolia, Tilia cordata, Salsapariglia root, Hop, Verbena officinalis, and Galega officinalis. The samples were prepared as reported in Section 3.1 and analyzed using the optimized MIPs-based procedure. To compare the performance, the samples have been analyzed in parallel with a method based on commercial IAC, which is commonly used for routine analysis. The IAC extraction was carried out according to the commercial protocol [33] (procedures reported in SI). This procedure was applied to analyze AFs in different samples such as spices, cereals, medicinal herbs, milk, and baby foods, and in all the cases recoveries between 60–90% were reported [34,35]. In general, the cleanup step using IAC allowed the purification and concentration of all samples before the analysis [36].

The recovery values (RC) and matrix effect (ME) obtained by fortifying the 17 samples at three levels (0.2, 5, and 10 µg/kg), using the MIPs-based and IAC procedures, are reported in Table 2. IAC gave lower recovery (50–80%) compared to the MIP-based approach (60–90%); moreover, the matrix effect was higher (IAC ≤ 30%; MIPs ≤ 15%). In both cases, good reproducibility was obtained (MIP procedure RSD ≤ 6, IAC procedure RSD ≤ 10). Considering the data obtained, the IAC-based procedure, despite the selectivity of the antibody, constantly displayed a higher ME for all foods tested with respect to MIP. Moreover, IAC procedure has a higher cost, the cleanup step takes a long time, and the required dilution of the sample leads to significant use of organic solvent (this sample dilution is needed to obtain selectivity). On the other hand, the MIP procedure is less affected by the matrix compounds and does not need sample dilutions; the eventual non-specific interactions generated by the matrix components are removed during the washing step.

Taking a look at the literature, there are some works where MIPs are synthesized for the selective extraction of AFB1 from food samples, and the mycotoxin is generally detected by HPLC–MS/MS. Díaz-Bao et al. [6] synthesized magnetic MIP stir bars using a classical synthesis (24 h) for the extraction of AFs in baby foods (wheat, corn, barley, and rice flour); quite good recoveries were reported (i.e., 39%, 44%, 40%, and 60% for the AFB1, AFB2, AFG1, and AFG2, respectively). Jayasinghe et al. [2] used a molecularly imprinted polymer for the selective micro-solid phase extraction and determination of aflatoxins; in this case, the method was applied just for fish feed, obtaining appreciable recoveries (>85%). Wu et al. [4] reported the use of SPE for AF extraction in grain using magnetized nanoporous carbon coated with MIPs, and in this case good recoveries were also obtained (70–90%). Considering the synthesis time, ease of production, low cost (including the dummy template), the ability to extract the 4 AFs, and the satisfactory analytical validation carried out, we are confident that the MIP-based procedure proposed here can become a valid and even more sustainable alternative for AFs analysis.

## 3. Materials and Methods

### 3.1. Chemicals, Reagents, and Samples

Aflatoxin B1, aflatoxin B2, aflatoxin G1, aflatoxin G2 from Aspergillus flavus ≥98% (HPLC), Citrinin (CIT) from Penicillium citrinum ≥98% (HPLC), Ochratoxin A, Ochratoxin B from Petromyces albertensis ≥98% (HPLC), Patulin ≥98.0% (HPLC), methacrylamide (MMA), acrylic acid (AA), methacrylic acid (MAA), 2-Vinylpyridine (2-VP), 1-Hydroxy-2-naphthoic acid (NH) ≥ 97%, ethylene glycol dimethacrylate (EGDMA), formic acid 99%, ammonium formate, and 2,2 azobis-isobutyronitrile (AIBN) were purchased from Sigma–Aldrich (Taufkirchen, Germany). Acetonitrile (ACN) 99.9%, ethanol 99.9%, hydrochloride acid 35%, dimethylsulfoxide (DMSO) 99%, acetone 99.8%, sodium hydroxide, dimethylformamide (DMF) 99%, and methanol 99.5% (MeOH) were purchased from VWR (Milano, Italy). The structures of the studied AFs and the components of the MIPs are shown in Figure S1.

Food supplements i.e., ginger, Echinacea purpurea, ginseng, hypericum, red elm, saffron, mango, red rice, parsley, red fruits, grapefruit, magnolia, Tilia cordata, Salsapariglia root, hop, Verbena officinalis, Galega officinalis were purchased from Sintal Dietetics s.r.l. (Castelnuovo Vomano, TE, Italy). The extraction of AFs from samples was performed according to Romero-Sánchez et al. [36] with some modifications. Briefly, 2.5 g of each sample was fortified and mixed with 5 mL of MeOH/H_2_O (80:20), vortexed for 5 min, and centrifuged for 15 min at 4500 rpm. Finally, the supernatant was filtered through a 0.2 µm PTFE syringe filter and stored at +4 °C before use. Before being loaded on the MIP-SPE, the extracts were diluted in water.

### 3.2. Apparatus

A probe sonicator Sonifier^®^ SFX550 (Branson, Danbury, CT, USA. Wattage Output: 550 W; frequency Output: 20 kHz) equipped with a Ø 13 mm disruptor horn was used for the synthesis of MIPs/NIPs. Precellys homogenizer (Bertin Technologies, Montigny-le-Bretonneux, France) was used for the crushing of MIPs and NIPs.

### 3.3. MIPs Synthesis

**Scheme 1:**
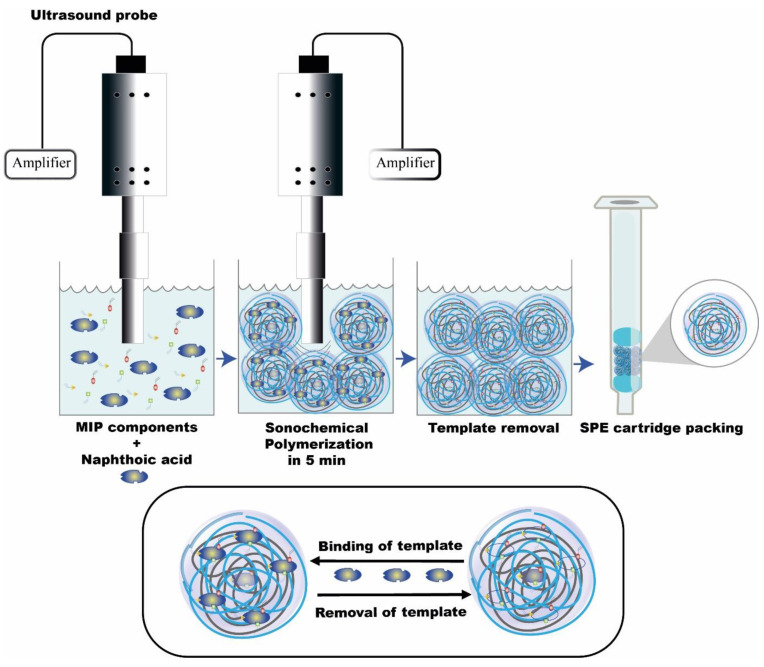
Graphical scheme of the ultrasound-probe-assisted MIP synthesis for AFs using a dummy template, and its use as solid-phase in SPE cartridge.

The MIP sonochemical synthesis is graphically resumed in Scheme 1. Briefly, the synthesis was performed as follows: the template NH (0.5 mmol/L) and functional monomer i.e., MMA (1.5 mmol/L) were dissolved in a 20 mL DMSO-water solution (50:50, *v/v*), and left under gentle mixing for 2 h. Then, the cross-linker EGDMA (5.19 mmol/L) and the initiator AIBN (0.12 mmol/L) were added, and the sonicator probe was placed two centimeters from the bottom of the beaker; the sonication was conducted at 50% of amplitude, in a continuous mode for 5 min. Afterwards, the MIP was washed with methanol:acetic acid (9:1, *v/v*) in a Soxhlet extractor; the template’s successful removal was confirmed by LC–MS/MS (see Section 3.6). The non-imprinted polymer (NIP) was prepared in the same way, except that no template was used.

The dry MIP and NIP were crushed and homogenized using a Precellys homogenizer (Bertin Technologies, Montigny-le-Bretonneux, France); 50 mg of MIP/NIP were placed in 7 mL plastic vials equipped with 10 ceramic microbeads (Ø = 1.3 mm), and the milling was performed at a speed of 5000 rpm for 10 s. The process was repeated 5 times.

### 3.4. MIP Binding Affinity Studies

The adsorption experiments were conducted using 5 mg of adsorbent phase (MIP or NIP), loading 1 mL of each AF (i.e., AFB1, AFB2, AFG1, AFG2) solution at different concentrations (from 2 to 250 ng/mL) using the extraction procedure reported in Section 3.5; LC–MS/MS was used to determine the retention of the AFs. The adsorption capacity Q (ng/mg) of the sorbent phase was calculated according to the following equation:

Q = [(Ci − Ce)/m] V

where Ci and Ce (µg·mL^−1^) are the initial and equilibrium concentration of the AFs, respectively. V (mL) is the volume of the solution and m (mg) is the amount of the MIP or NIP. The equilibrium data were fitted using non-linear Freundlich and Langmuir models, and the corresponding equations are summarized in Table S6.

The imprinting factor (IF) reflects the efficiency of the MIP over its NIP. When the IF is higher than one, the template-shaped cavities in the polymer matrix are successfully created; a higher IF means higher effectiveness of MIP.

It was calculated according to the following equation:

IF = QMIP/QNIP

where QMIP and QNIP are the binding capacities of MIP and NIP, respectively.

### 3.5. Sample Cleanup

**Scheme 2:**
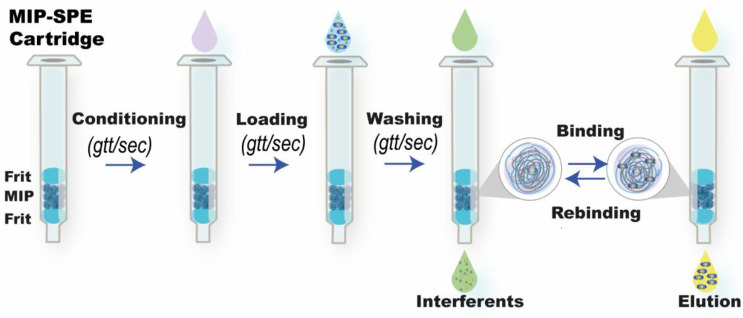
Graphical scheme of the solid-phase extraction procedure based on the cartridge containing the MIP.

A solid-phase extraction (SPE) procedure was developed using the synthesized MIPs as the sorbent phase (MIP–SPE): 5 mg of MIP (or NIP) were placed in a 1 mL empty SPE-cartridge between two polyethylene frits. The procedure relies on four easy steps that are graphically reported in Scheme 2: (i) the cartridge is conditioned with 1 mL of water, (ii) the AFs-standard (or sample extract) is loaded in water, (iii) the cartridge is then washed with 1 mL of 0.5% ACN in water, and finally (vi) the elution is performed with 1 mL of 2% acetic acid in MeOH solution.

For IAC, the procedure was reported in the Supplementary Materials.

### 3.6. LC–MS/MS

All analyses were performed on a Nexera LC20AD XR system from Shimadzu (Kyoto, Japan), with Prominence 20AD autosampler. The system was equipped with a vacuum degasser and column oven coupled with a 4500 Qtrap from Sciex (Toronto, ON, Canada) equipped with a Turbo V electrospray ionization (ESI) source.

For analysis, an ACE Excel 2 C18-PFP (10 cm × 2.1 mm id) from Advanced Chromatography Technologies (Aberdeen, Scotland, UK) packed with particles of 2 μm, equipped with a security guard column, was used. The mobile phase consisted of water containing 5 mM ammonium formate (phase A) and MeOH/CAN 50:50 with 5 mM formic acid (phase B). Analysis was performed using the following gradient elution at a flow rate of 0.3 mL min^−1^: from 0 to 0.3 min gradient was held at 80% of phase B; from 0.3 to 3 min phase B was decreased from 80% to 75%; from 3 to 3.5 min was increased from 75% to 99%; to 3.5 min the concentration of phase B was 99% and was held for 3 min. Gradient returned to initial condition in 0.2 min, followed by a 3 min equilibration, in a total run time of 9.5 min. The injection volume was 3 µL and all samples were injected in triplicate.

The analytes were detected in ESI positive mode except for Patulin which was detected in ESI negative mode with a capillary voltage of +5500 V, using air ion source gas at 60 psi and nitrogen ion source gas at 40 psi at a temperature of 500 °C. Two multi-reaction monitoring (MRM) transitions were chosen for each analyte. All source and instrument parameters for the monitored analytes were tuned by injecting every single standard solution at a concentration of 10 ng/µL at 7 µL/min by a syringe pump. The ion currents were acquired in MRM mode and quantitation was performed using Multiquant Software from Sciex (Concord, ON, Canada). All samples were analyzed in triplicate. The selected MRM transitions and HPLC–MS/MS parameters are reported in Table S7.

### 3.7. Method Validation

The proposed method was validated on the four target analytes (AFB1, AFB2, AFG1, and AFG2) according to the FDA guidelines [37], considering the following parameters: limit of detection (LOD), limit of quantification (LOQ), linearity, precision, and accuracy. For the linear dynamic range, a calibration curve consisting of six points was built and each point was performed in triplicate. The linearity was checked between LLOQs and 50 ng mL^−1^ for all analytes. The LLODs and LLOQs were estimated at a signal-to-noise (S/N) ratio of three and ten, respectively. Accuracy was determined by spiking the analytes in matrices in triplicate for three different levels of concentration. For precision determination intraday RSDs were considered one-day measures of three sample replicates at three levels of concentration (intra-day precision), whereas for inter-day RSDs, samples were analyzed for three consecutive days and each day was analyzed twice.

## 4. Conclusions

In this work, we successfully proposed a rapid and affordable method to synthesize MIPs for AFs extraction from different food matrices. The MIP was used as sorbent phase in SPE cartridges and coupled to LC–MS/MS determination. Despite the heterogeneity of the samples tested, the proposed MIPs-based procedure showed appreciable recoveries (65–90%; RSD < 6%, n = 3) and a low matrix effect (ME < 15%), resulting in better performance when compared to the immunoaffinity column-based commercial method. The proposed method is rapid, does not need organic solvents, and presents reduced costs with respect to commercial dedicated cartridges for AFs extraction. In summary, this work demonstrates how a straightforward sonochemical and fast-synthesis approach allows the production of effective MIPs that are able to selectively extract AFs from different complex food matrices.

## Figures and Tables

**Table 1:** Adsorption isotherms parameters obtained for AFs onto MIP and NIP.

	Models	Parameters	MIP	NIP
AFB1	Freundlich	R^2^	0.95	0.91
		KF (ng/mg)	13.22	2.57
		nF	2.37	2.71
	Langmuir	R^2^	0.99	0.98
		Qmax (ng/mg)	50.46	16.72
		Kl (mL/ng)	0.53	0.07
AFB2	Freundlich	R^2^	0.94	0.91
		KF (ng/mg)	12.35	2.55
		nF	2.33	2.70
	Langmuir	R^2^	0.99	0.98
		Qmax (ng/mg)	49.94	16.78
		Kl (mL/ng)	0.49	0.07
AFG1	Freundlich	R^2^	0.94	0.90
		KF (ng/mg)	12.88	0.52
		nF	2.46	1.61
	Langmuir	R^2^	0.98	0.94
		Qmax (ng/mg)	49.47	27.03
		Kl (mL/ng)	0.52	0.01
AFG2	Freundlich	R^2^	0.94	0.94
		KF (ng/mg)	13.12	1.06
		nF	2.57	2.03
	Langmuir	R^2^	0.99	0.91
		Qmax (ng/mg)	48.21	17.35
		Kl (mL/ng)	0.59	0.02

**Table 2:** Data obtained analyzing food samples using the MIPs-based and IAC-based procedures.

Sample	AFG1				AFG2				AFB1				AFB2			
	MIPs–SPE (%)		IAC (%)		MIPs–SPE (%)		IAC (%)		MIPs–SPE (%)		IAC (%)		MIPs–SPE (%)		IAC (%)	
	RC	ME	RC	ME	RC	ME	RC	ME	RC	ME	RC	ME	RC	ME	RC	ME
Ginger	60	+9	50	−30	63	−6	48	+28	64	−1	50	+25	81	−14	52	+30
Echinacea purpurea	83	+9	73	+15	66	+10	75	+15	90	−1	80	+10	60	−8	72	+12
Ginseng	75	+16	66	−26	60	+1	46	−23	90	+7	67	−28	78	+15	61	−29
Hypericum	50	+15	50	27	60	+15	38	+30	53	+11	52	+25	72	+1	50	+30
Red elm	77	+10	64	+5	70	+7	63	+15	90	−4	80	+10	74	−6	80	+8
Saffron	61	+16	60	+12	69	+11	62	+20	60	+15	60	+17	68	+9	64	+15
Mango	67	+10	65	+15	65	+7	60	+15	65	+10	70	+15	65	+10	68	+12
Red rice	76	+11	50	+25	65	+15	55	+20	89	+3	65	+20	70	+9	66	+20
Parsley	60	+9	50	+30	60	+10	40	+30	76	+1	45	+28	60	+4	43	+19
Red fruits	60	+7	55	+20	60	+10	56	+21	79	+6	60	+18	60	+10	58	+22
Grapefruit	61	+11	65	+15	69	+11	70	+12	68	+12	70	+15	71	+4	68	+16
Magnolia	61	−7	50	+30	64	+12	50	+20	64	+15	50	+18	77	+8	54	+20
Tilia cordata	60	+1	50	+20	62	+12	45	+18	62%	+11	50	+19	72	+2	50	+20
Salsapariglia root	62	+3	55	+20	67	+6	52	+18	72	+15	50	+20	69	+14	70	+17
Hop	60	+2	50	+20	72	+12	51	+20	74	+15	60	+17	57	+12	67	+18
Verbena officinalis	72	+5	56	+18	60	+15	55	+21	69	−8	67	+18	65	+13	66	+16
Galega officinalis	78	+4	64	+15	77	+17	65	+20	73	+14	65	+20	75	+3	74	+20

## Data Availability

All data are included within the article and Supplementary Materials.

## Supplementary Materials

The following supporting information can be downloaded at: https://www.mdpi.com/article/10.3390/molecules28020703/s1, Table S1: Comparative table summarizing the literature works related to MIPs-SPE for AFs using HPLC-MS as detection method; Figure S1: Structures of aflatoxin B1, aflatoxin B2, aflatoxin G1, aflatoxin G2, 1-hydroxy-2-napthoic acid, methacrylamide (MMA), ethylene glycol dimethacrylate (EGDMA) and 2,2 azobis (isobutyronitrile) (AIBN); Table S2: Data obtained for the MIPs-SPE procedure optimization; Figure S2: Adsorption isotherms models for the adsorption of AFs onto MIP and NIP; Table S3: Lower limit of quantification (LLOQ), lower limit of detection L(LOD), calibration curve equation and determination coefficient obtained in analytical procedure validation; Table S4: Accuracy of the result obtained in the validation reported as BIAS percentage (%); Table S5: Intra-day and inter-day precision reported in percentage (%); Table S6: The isotherm equations; Table S7: MS/MS parameters; IAC extraction procedure.

## Sample Availability

Samples of the compounds are available from the authors.
