# Supplementary material for: Study on Molecularly Imprinted Polymers Obtained Sonochemically for the Determination of Aflatoxins in Food
Source: Molecules. 2023 Jan 10;28(2):703. doi: 10.3390/molecules28020703 (PMC9861586; doi:10.3390/molecules28020703)
Supplement: Supplementary file 1 [file molecules-28-00703-s001.zip › molecules-2097846-supplementary.pdf]

SUPPLEMENTARY MATERIAL

Article

Study on Molecularly Imprinted Polymers Obtained Sonochemically for the Determination of Aflatoxins in Food

Sara Palmieri [1,†], Dounia Elfadil [1,2,†], Federico Fanti [1], Flavio Della Pelle [1], Manuel Sergi [1], Aziz Amine [2,*] and Dario Compagnone [1,*]

1 Department of Bioscience and Technology for Food, Agriculture and Environment, University of Teramo, via Renato Balzarini 1, 64100 Teramo, Italy
2 Laboratory of Process Engineering and Environment, Faculty of Sciences and Techniques, Hassan II University of Casablanca, Mohammedia 20650, Morocco
* Correspondence: a.amine@univh2m.ac.ma (A.A.); dcompagnone@unite.it (D.C.)
† These authors contributed equally to this work.

Table S1. Comparative table summarizing the literature works related to MIPs-SPE for AFs using HPLC-MS as detection method.

Target	Monomer	Dummy template	Method/Time of synthesis	Selectivity	LOD	Real sample	REF
Aflatoxins B1, B2, G1, and G2	MAA	DMC (5,7- dimethoxycoumarin)	precipitation polymerization/26 h.	-β Carotein<br>-Carboxymethyl cellulose<br>- carrageenan (kappa and lambda)<br>-Agar-agar<br>-Vit A<br>-Vit D	AFB1LOD=0.42 μg /kg<br>AFB2LOD=0.88 μg /kg<br>AFG1LOD=1.1 μg /kg<br>AFG2LOD=1.2 μg /kg	Fish feed	[1]
Aflatoxins B1, B2, G1, and ,M1	APTES and TEOS	DMC (5,7- dimethoxycoumarin)	-Sol-gel method/ 16 h.	Ochratoxin, patulin, Zearalenon, Fumonisin B1, Coumarin, Bisphenol-A, Chlorpyrifos, Cypermethrin, diazinon, dichlorvos, and malathion, with a concentration of 10 ng mL− 1 and glucose, lactose, fructose, sucrose, vitamin C, cysteine, serine, histidine, and urea with a concentration of 2 μg mL− 1	AFB1LOD=0.021 ng /mL<br>AFB2LOD=0.024 ng /mL<br>AFG1LOD=0.023 ng /ml<br>AFM1LOD= 0.026 ng /mL	Rice; Wheat Flour and Milk.	[2]
Aflatoxins B1, B2 and G1.	MAA	ethyl 3- coumarincarboxylate	- Atom transfer radical precipitation polymerization (ATRPP)/8 h.	Ochratoxin and Zearalenon.	AFB1LOD= 0.05ng /mL<br>AFB2LOD= 0.05 ng /mL<br>AFG1LOD= 0.07 ng /ml	spiked corn	[3]
Aflatoxins B1, B2 G1, G2 and M1.	MAA	5,7-Dimethoxycoumarin (DMC)	Bulk polymerization/24h	-	AFB1LOD=0.9 ng /Kg<br>AFB2LOD=0.7 ng /Kg<br>AFG1LOD=1.0 ng /Kg<br>AFG2LOD=1.7 ng /Kg<br>AFM1LOD= 0.3 ng /Kg	Baby food	[4]
Aflatoxins B1, B2, G1, and G2	Acrylamide	Quercetin	precipitation polymerization/7 h.	Zearalenone, coumarin and isoxepac.	AFB1LOD=0.09ng/mL<br>AFB2LOD=0.13ng/mL<br>AFG1LOD=0.10ng /ml<br>AFG2LOD=0.06ng/mL	Wheat, Soybean,rice and corn.	[5]
Aflatoxins B1, B2, G1, and G2	Methacrylamide	Naphtoic acid	Free radical polymerization/5min	Citrinin,patulin,ochratoxin A and ochrathoxin B.	AFB1LOD=0.005ng/mL<br>AFB2LOD=0.027ng/mL<br>AFG1LOD=0.007ng/ml<br>AFG2LOD=0.009ng/mL	Ginger, Echiancea purpurea, Ginseng, Hypericum, Red elm, Saffron Mango, Red rice, Parsley, Red fruits, Grapefruit, Magnolia, Tilia Cordata, Root Salsopariglia Hop, Verbene Officinalis, Galega Officinalis.	This work

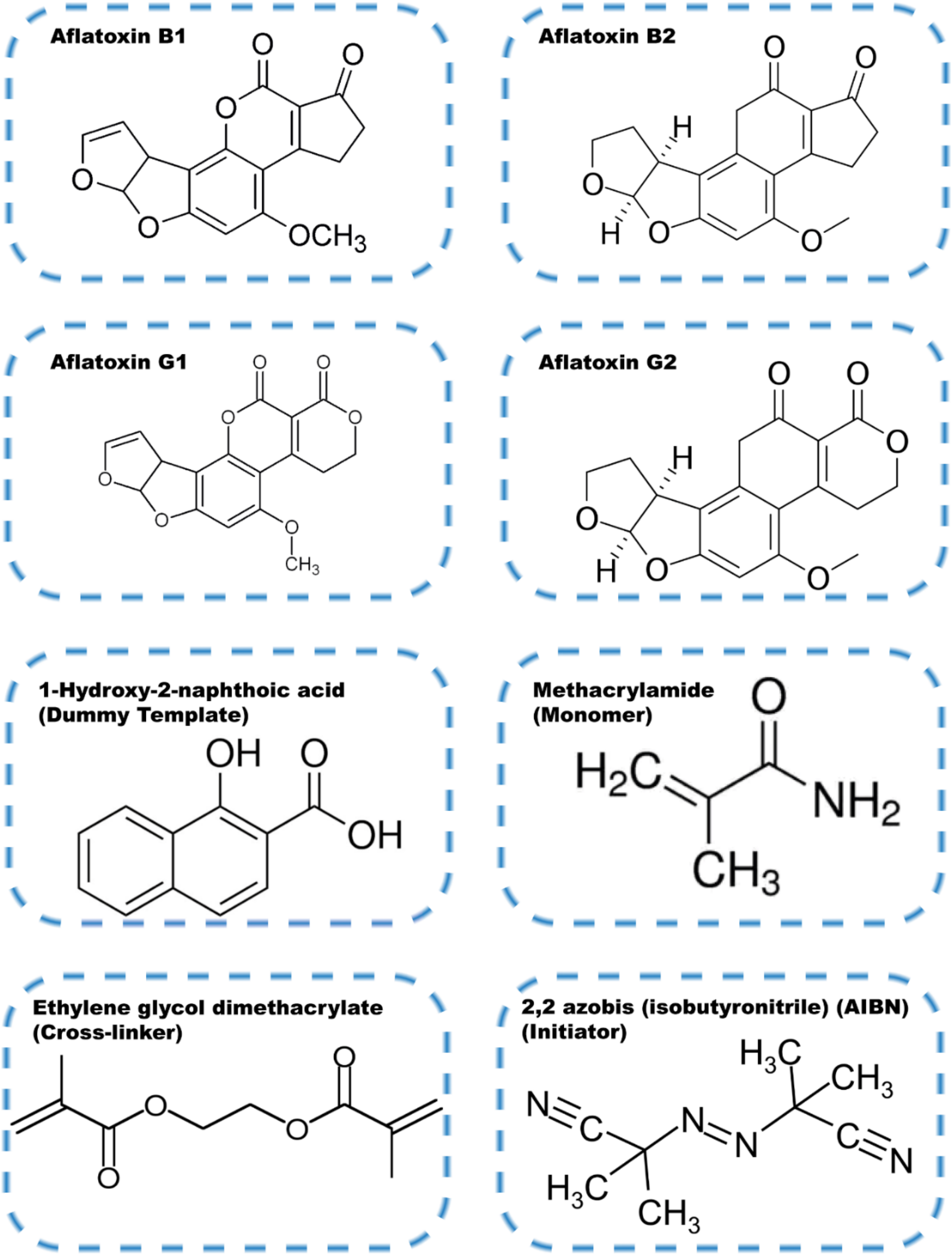

Figure S1. Structures of aflatoxin B1, aflatoxin B2, aflatoxin G1, aflatoxin G2, 1-hydroxy-2-napthoic acid, methacrylamide (MMA), ethylene glycol dimethacrylate (EGDMA) and 2,2 azobis (isobutyronitrile) (AIBN).

Table S2. Data obtained for the MIPs-SPE procedure optimization; the values are expressed as % of AFs adsorbed (A and B) and released from the MIP. In red are reported the chosen parameters. 5 ppb of AFs were employed for all the tests performed.

**Amount of Polymer**				
	AFB1 (%)	AFB2 (%)	AFG1 (%)	AFG2 (%)
2 mg	50	55	60	57
5 mg	85	85	90	90
10 mg	85	85	90	90
20 mg	85	85	90	90
**Washing**				
water	8	10	7	5
(80:20) ACN:H20	70	70	48	50
0.5%ACN H20	4	2	2	3
5% ACN H20	40	31	39	42
1% ACN H20	25	20	17	27
**Elution**				
MeOH	63	64	65	64
2% acetic acid in MeoH	85	85	90	90

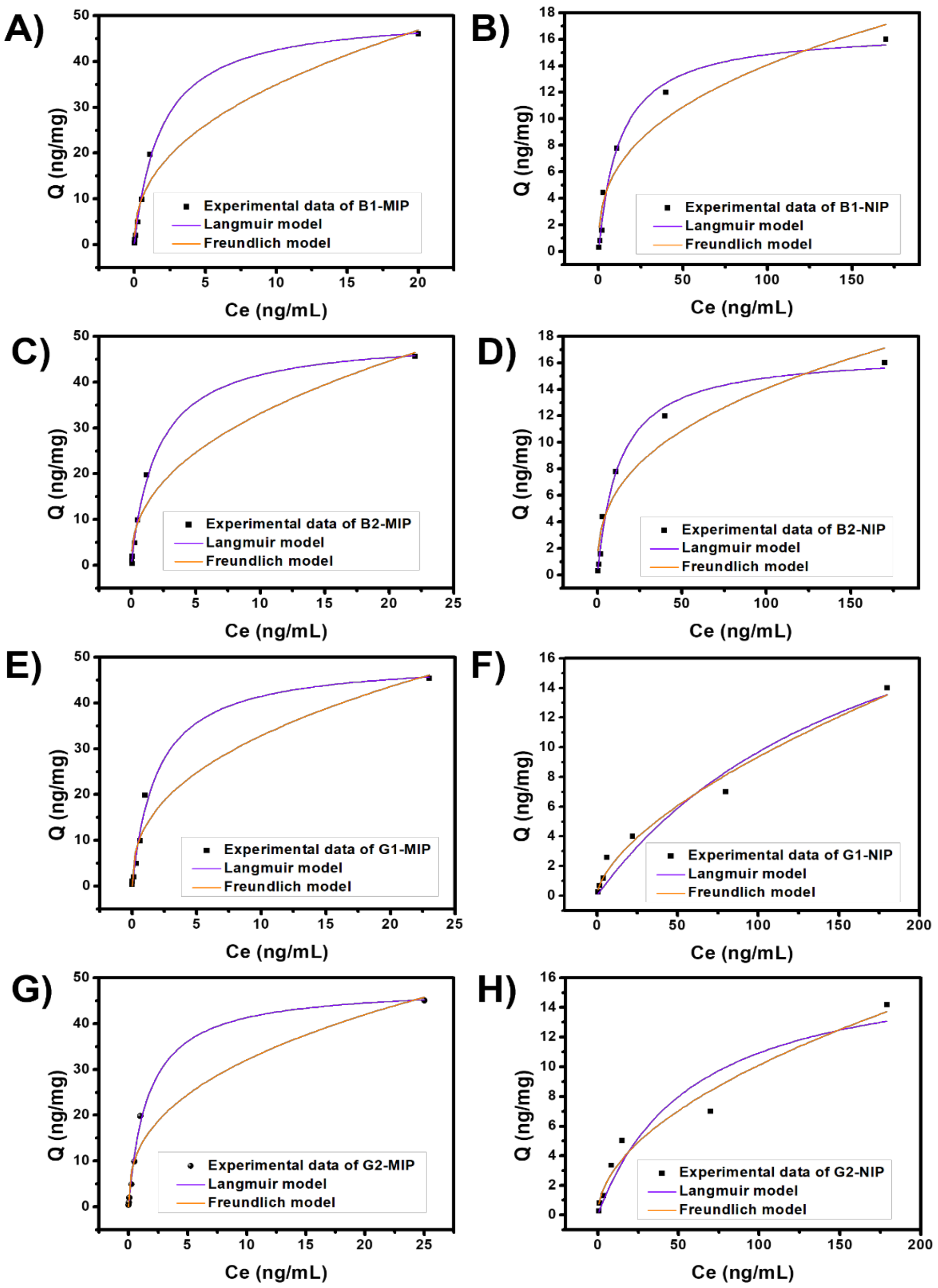

Figure S2. Adsorption isotherms models for the adsorption of AFs onto MIP and NIP. A, C, E, and G represent the experimental data of adsorption by MIP at different concentrations at room temperature and the corresponding adsorption isotherms models including Langmuir and Freundlich models for AFB1, AFB2, AFG1, AFG2, respectively. B, D, F, and H represent the experimental data of adsorption by NIP at different concentrations at room temperature and the corresponding adsorption isotherms models including Langmuir and Freundlich models for AFB1, AFB2, AFG1, AFG2, respectively.

Table S3. Lower limit of quantification (LLOQ), lower limit of detection L(LOD), calibration curve equation and determination coefficient obtained in analytical procedure validation.

**Analyte**	**LLOQ (ng mL^{-1})**	**LLOD (ng mL^{-1})**	**Calibration Curve**	**R^2**
AFG1	0.02	0.005	y = 5696x - 1021	0.996
AFG2	0.09	0.027	y = 1683x - 3697	0.999
AFB1	0.02	0.007	y = 2875x - 6827	0.999
AFB2	0.03	0.009	y = 1889x - 1519	0.997

Table S4. Accuracy of the result obtained in the validation reported as BIAS percentage (%).

	Accuracy (BIAS %)											
	AFG1			AFG2			AFB1			AFB2		
	0.2 µg/kg	5µg/kg	10 µg/kg	0.2 µg/kg	5 µg/kg	10 µg/kg	0.2 µg/kg	5 µg/kg	10 µg/kg	0.2 µg/kg	5 µg/kg	10 µg/kg
Ginger	+4%	+3%	+3%	+4%	+3%	+3%	+4%	+4%	+4%	+3.0%	+3%	+4%
Echiancea purpurea	+3%	+3%	+4%	+3%	+3%	+4%	+4%	+3%	+4%	+4%	+3%	+5%
Ginseng	+4%	+3%	+5%	+3%	+3%	6%	+4%	+4%	+2%	+4%	+3%	+4%
Hypericum	+4%	+5%	+5%	+3%	+4%	+6%	+4%	+3%	+3%	+4%	+4%	+5%
Red elm	+4%	+5%	+5%	+4%	+2%	+3%	+4%	+4%	+3%	+4%	+2%	+4%
Saffron	+4%	+3%	+4%	+4%	+4%	+3%	+4%	+2%	+5%	+4%	+4%	+5%
Mango	+4%	+4%	+5%	+4%	+3%	+5%	+4%	+3%	+4%	+4%	+3%	+4%
Red rice	+4%	+5%	+4%	+4%	+3%	+4%	+4%	+3%	+5%	+4%	+4%	+5%
Parsley	+4%	+4%	+4%	+4%	+5%	+3%	+4%	+5%	+5%	+4%	+5%	+5%
Red fruits	+3%	+3%	+5%	+4%	+5%	+4%	+4%	+3%	+5%	+4%	+5%	+5%
Grapefruit	+4%	+5%	+5%	+3%	+3%	+5%	+4%	+3%	+4%	+2%	+3%	+5%
Magnolia	+3%	+5%	+5%	+3%	+3%	+6%	+4%	+4%	+5%	+4%	+5%	+5%
Tilia Cordata	+4%	+4%	+5%	+4%	+4%	+6%	+4%	+4%	+5%	+4%	+5%	+4%
Root Salsopariglia	+3%	+5%	+4%	+3%	+5%	+4%	+4%	+4%	+3%	+3%	+5%	+5%
Hop	+3%	+4%	+4%	+4%	+5%	+6%	+5%	+3%	+5%	+3%	+5%	+3%
Verbene Officinalis	+3%	+5%	+5%	+3%	+5%	+6%	+4%	+4%	+5%	+5%	+4%	+5%
Galega Officinalis	+4%	+4%	+5%	+3%	+4%	+4%	+4%	+4%	+5%	+5%	+4%	+5%

Table S5. Intra-day and inter-day precision reported in percentage (%).

	**Precision (RSD %)**																							
	AFG1						AFG2						AFB1						AFB2					
	Intra-day			Inter-day			Intra-day			Inter-day			Intra-day			Inter-day			Intra-day			Inter-day		
	0.2 µg/kg	5 µg/kg	10 µg/kg	0.2 µg/kg	5 µg/kg	10 µg/kg	0.2 µg/kg	5 µg/k	10 µg/kg	0.2 µg/kg	5 µg/kg	10 µg/kg	0.2 µg/kg	5 µg/kg	10 µg/kg	0.2 µg/kg	5 µg/kg	10 µg/kg	0.2 µg/kg	5 µg/kg	10 µg/kg	0.2 µg/kg	5 µg/kg	10 µg/kg
Ginger	2%	3%	2%	2%	4%	4%	4%	3%	3%	4%	4%	4%	4%	3%	4%	4%	4%	4%	4%	2%	4%	3%	4%	3%
Echiancea purpurea	3%	3%	3%	3%	3%	2%	2%	3%	4%	2%	2%	4%	3%	2%	3%	4%	3%	4%	5%	3%	4%	4%	4%	4%
Ginseng	2%	3%	3%	3%	3%	4%	3%	3%	4%	3%	4%	2%	4%	1%	4%	4%	2%	3%	4%	3%	4%	3%	3%	3%
Hypericum	3%	3%	4%	2%	4%	4%	3%	4%	2%	3%	3%	3%	5%	4%	5%	2%	3%	4%	5%	3%	2%	4%	4%	4%
Red elm	3%	3%	4%	4%	4%	4%	2%	2%	3%	2%	4%	3%	4%	2%	4%	3%	3%	5%	5%	4%	3%	2%	3%	3%
Saffron	2%	3%	2%	3%	4%	3%	4%	4%	3%	4%	2%	2%	4%	4%	4%	3%	2%	4%	4%	2%	3%	3%	3%	4%
Mango	3%	4%	4%	3%	4%	3%	4%	3%	2%	4%	3%	4%	4%	3%	4%	2%	4%	5%	2%	2%	2%	3%	3%	4%
Red rice	4%	5%	5%	3%	3%	3%	4%	3%	4%	3%	3%	3%	2%	4%	3%	5%	3%	5%	3%	4%	4%	4%	3%	4%
Parsley	7%	4%	4%	2%	7%	5%	5%	7%	3%	3%	5%	4%	3%	5%	4%	5%	5%	2%	3%	3%	2%	4%	5%	4%
Red fruits	4%	3%	5%	2%	4%	3%	4%	4%	4%	2%	3%	5%	3%	5%	3%	4%	5%	3%	2%	4%	3%	2%	4%	5%
Grapefruit	5%	4%	5%	3%	4%	3%	4%	3%	5%	3%	3%	4%	2%	3%	3%	3%	4%	3%	4%	3%	3%	3%	4%	5%
Magnolia	5%	5%	5%	3%	3%	3%	2%	3%	4%	3%	4%	2%	4%	4%	2%	4%	4%	2%	3%	4%	2%	3%	4%	3%

Tilia Cor-data	5%	4%	5%	2%	3%	6%	4%	4%	4%	2%	4%	3%	4%	4%	4%	5%	4%	2%	4%	4%	3%	3%	5%	4%
Root Salsop ariglia	4%	5%	5%	4%	5%	4%	3%	4%	4%	4%	4%	3%	3%	5%	2%	4%	5%	3%	5%	5%	3%	5%	4%	4%
Hop	5%	4%	5%	4%	5%	4%	2%	4%	5%	5%	3%	2%	3%	5%	3%	5%	4%	3%	4%	5%	2%	5%	5%	4%
Ver-bene Offici-nalis	5%	5%	4%	4%	5%	3%	3%	5%	4%	4%	4%	4%	5%	4%	3%	5%	4%	2%	5%	5%	5%	4%	4%	4%
Galega Offici-nalis	5%	4%	5%	4%	4%	3%	3%	4%	4%	4%	4%	4%	5%	4%	2%	4%	5%	5%	4%	5%	4%	5%	5%	4%

Table S6. The isotherm equations

Model	Equation	Parameters
Langmuir	$Q_e = \frac{Q_{max} K_L C_e}{1 + C_e}$	k_L (mL/ng) is the Langmuir constant; Q_{max} (ng/mg) represents the maximum adsorption capacity obtained with Langmuir model.
Freundlich	$Q_e = K_F C_e^{1/n}$	k_F (ng/mg) and n_F are the Freundlich model constants.

Table S7. MS/MS parameters. Multi-reaction monitoring (MRM) transitions: Q1 MASS = precursor ion mass (amu); Q3 MASS = product ion mass (amu). TIME = dwell time. DP (V) = declustering potential (amu). EP (V) = entrance potential. CE (V) = collision energy. CXP (V) = cell exit potential.

Analytes	Q1	DP	EP	DT	Q3	CE	CXP
MRM (+)							
Aflatoxin G1	329.1	110	10	50	242.9	36	9
					310.9	31	10
Aflatoxin G2	331.1	110	8	50	313.1	35	18
					245.1	43	14
Aflatoxin B1	313.1	107	7	50	285.0	32	11
					241.0	50	9
Aflatoxin B2	315.1	100	9	50	287.1	36	9
					258.8	40	11
Ochratoxin A	404.3	81	9	50	358.1	21	15
					239.1	32	9
Aflatoxin M1	329.2	96	7	50	273	35	10
					258.7	33	12
Citrinina	251.0	55	13	50	233.0	24	9
					205.0	36	7
Ochratoxin B	370.2	50	7	25	205.0	23	12
					324.0	20	12
MRM (-)							
Patulina I	153.0	-50	-12	50	109.0	-13	-7
					81.0	-17	-5

IAC Procedure

The AFs extraction via IAC was performed as follow: 25 g of sample were extracted by adding 5g of NaCl and 100 mL of MeOh:H_2O 80:20 v/v; the extraction was performed shaking at high speed with a magnet for 2 min; then the sample was centrifuged at 4000 g for 10 min.

A 4 mL aliquot was diluted with 36 mL of PBS solution and then filtered; 20 mL of filtered sample was loaded on IAC at flow rate of 2 mL min^{-1}; the washing step was performed with 20 mL of H_2O at flow rate of 5 mL min^{-1}; finally the elution of the target analytes was achieved with 1 mL of MeOH and subsequently 1 mL of H_2O; 100 μL of elution was analyzed by HPLC-MS/MS (see section 2.6).

References

1. Jayasinghe, G.D.T.M.; Domínguez-González, R.; Bermejo-Barrera, P.; Moreda-Piñeiro, A. Ultrasound assisted combined molecularly imprinted polymer for the selective micro-solid phase extraction and determination of aflatoxins in fish feed using liquid chromatography-tandem mass spectrometry. Journal of Chromatography A 2020, 1609, 460431, doi:https://doi.org/10.1016/j.chroma.2019.460431.
2. Alilou, S.; Amirzehni, M.; Eslami, P.A. A simple fluorometric method for rapid screening of aflatoxins after their extraction by magnetic MOF-808/graphene oxide composite and their discrimination by HPLC. Talanta 2021, 235, 122709, doi:https://doi.org/10.1016/j.talanta.2021.122709.
3. Wu, C.; He, J.; Li, Y.; Chen, N.; Huang, Z.; You, L.; He, L.; Zhang, S. Solid-phase extraction of aflatoxins using a nanosorbent consisting of a magnetized nanoporous carbon core coated with a molecularly imprinted polymer. Mikrochimica acta 2018, 185, 515, doi:10.1007/s00604-018-3051-8.
4. Díaz-Bao, M.; Regal, P.; Barreiro, R.; Fente, C.A.; Cepeda, A. A facile method for the fabrication of magnetic molecularly imprinted stir-bars: A practical example with aflatoxins in baby foods. Journal of Chromatography A 2016, 1471, 51-59, doi:https://doi.org/10.1016/j.chroma.2016.10.022.
5. Liang, Y.; He, J.; Huang, Z.; Li, H.; Zhang, Y.; Wang, H.; Rui, C.; Li, Y.; You, L.; Li, K.; et al. An amino-functionalized zirconium-based metal-organic framework of type UiO-66-NH(2) covered with a molecularly imprinted polymer as a sorbent for the extraction of aflatoxins AFB1, AFB2, AFG1 and AFG2 from grain. Mikrochimica acta 2020, 187, 32, doi:10.1007/s00604-019-3959-7.
